# Tackling global inequalities in maternal hypertensive disorders: trends and the impact of public health emergencies, 1990–2021

**DOI:** 10.3389/fpubh.2025.1696754

**Published:** 2025-10-13

**Authors:** Siying Wei, Junliang Zhu, Changyu Ni, Jianing Bi, Zhongxun Dong, Xueying Xu, Weijie Cai, Xiaojin Wang, Hongbo Liu, Bingshun Wang

**Affiliations:** ^1^School of Public Health, Shanghai Jiao Tong University School of Medicine, Shanghai, China; ^2^Department of Biostatistics, Clinical Research Institute, Shanghai Jiao Tong University School of Medicine, Shanghai, China; ^3^Department of Health Statistics, School of Public Health, China Medical University, Shenyang, China; ^4^Key Laboratory of Environmental Stress and Chronic Disease Control and Prevention, Ministry of Education, China Medical University, Shenyang, China; ^5^Clinical Research Center, Ruijin Hospital Luwan Branch, School of Public Health, Shanghai Jiao Tong University School of Medicine, Shanghai, China

**Keywords:** global burden of disease, inequality, public health emergency, socio-demographic index, maternal health, epidemiology, maternal hypertensive disorders

## Abstract

**Background and aims:**

Despite progress made under the United Nations Millennium Development Goals (MDGs) and the Sustainable Development Goals (SDGs), inequalities in global health persist, particularly in the maternal health. Public health emergencies also affect health equity. This research examined long-term disease burden trends (1990–2021) of maternal hypertensive disorders (MHD), with a focus on the influence of age and socio-demographic index (SDI) differences, as well as short-term disruptions during the COVID-19 pandemic, to inform more equitable maternal health policies.

**Methods:**

Using the Global Burden of Disease database (2021), this study conducted a systematic examination of indicators of MHD: incidence, maternal mortality ratio (MMR), and disability-adjusted life years (DALYs). All analyses were standardized for age-specific fertility rates (ASFR). Analyses included frontier analysis to identify achievable health outcomes, decomposition analysis to identify key factors, and age-period-cohort (APC) model to assess independent effects. Health inequalities were measured using the slope index of inequality (SII) and concentration curve. The secular trends were characterized using the average annual percentage change (AAPC), while the impact of the COVID-19 pandemic was assessed through the estimated annual percentage change (EAPC).

**Results:**

The APC model revealed elevated risks for adolescent and older pregnancies, with incidence fluctuating over 32 years but MMR steadily declining. Disease burden generally decreased with higher SDI levels. Decomposition analysis suggested that demographic factors increased the burden, while epidemiology mitigated it. Frontier analysis indicated effective maternal health control in Canada but Cameroon required progress. While the SII in 2021 declined [−2003.35 (95% CI: −2184.75 to −1821.96)], concentration curves revealed increased relative inequality in lower-SDI populations. Over 32 years, the global MHD burden declined, with a reduction in low-SDI regions [AAPC: −29.46 (95% CI: −30.06 to −28.85)] approximately 24 times that of high-SDI regions. However, the pandemic significantly slowed the decline in low- and low-middle SDI regions.

**Conclusion:**

This study highlights marked disparities in the disease burden among age groups across diverse SDI regions. Public health emergencies have intensified existing health inequalities and exposed gaps in healthcare resource distribution. Implementing targeted interventions and reinforcing maternal care during emergencies are critical for enhancing maternal health and advancing health equity.

## 1 Introduction

Health disparities across regions remain a critical challenge in global health. Inequalities in economic development, healthcare accessibility, and educational levels contribute to uneven health outcomes around the world (1). Maternal health remains a fundamental component of the global health agenda, serving as a vital indicator of population well-being, social progress, and public health advancement (2). Ensuring equitable maternal health care not only addresses a vulnerable population’s needs but also reflects the broader progress toward achieving global health equity and sustainable development. The Millennium Development Goals (MDGs) called for significant reductions in maternal mortality and expanded access to reproductive health services. Despite some progress from 2000 to 2015, maternal health outcomes remain highly unequal (3). These disparities have continued under the Sustainable Development Goals (SDGs) (4). Maternal hypertensive disorders (MHD) comprise a group of serious pregnancy-related conditions, affecting an estimated 7.3% of pregnant women worldwide (5) and accounting for 14% of maternal deaths during childbirth (6). Alarmingly, the prevalence of MHD has risen by 25% over the past decade (7), leaving modern mothers more vulnerable than their predecessors, even after accounting for factors such as advanced maternal age (8). Moreover, pronounced disparities in healthcare accessibility exacerbate this issue, disproportionately elevating the risk of MHD among vulnerable populations of women (9). This escalating crisis necessitates urgent action.

Since the beginning of the 21st century, human society has been confronted with two major public health crises caused by highly pathogenic beta-coronaviruses. In late 2002 to early 2003, the emergence of SARS-CoV led to the outbreak of severe acute respiratory syndrome (SARS), which spread to nearly 30 countries and regions worldwide. At the end of 2019, the outbreak of SARS-CoV-2 triggered the global pandemic of COVID-19, which in terms of geographic spread, number of infections, and socioeconomic impact, far exceeded that of SARS (10). As a significant international public health emergency, the emergence of the COVID-19 pandemic placed unprecedented pressure on global healthcare systems. The strain on medical resources, deterioration of socioeconomic conditions, and disruption of maternal healthcare services severely affected the early detection and management of pregnancy-related complications (11). Alarmingly, pregnant women infected with SARS-CoV-2 were found to face an elevated risk of preterm birth and often require higher levels of intensive care (12). Against this backdrop, this study, utilizing GBD data, provided the first systematic assessment of the short-term impact of public health emergencies on the global disease burden of MHD, with a focus on the case of the COVID-19 pandemic.

Global health inequities manifest in maternal health through significant variations among different age groups across regions. This study presented a comprehensive analysis of GBD data from 1990 to 2021, aiming to offer evidence-based insights to inform targeted interventions that address health disparities and strengthen maternal healthcare delivery across diverse populations.

## 2 Methods

### 2.1 Data collection

The Global Burden of Disease Study 2021 (GBD 2021) provides data from the Global Health Data Exchange^1^ on 371 diseases across 204 countries and territories from 1990 to 2021. Methodological details and modeling strategies for GBD 2021 have been thoroughly documented in previous publications by the research team (13). Incidence, MMR, and DALYs for MHD, along with their 95% uncertainty intervals (95% UIs), were analyzed by location (204 countries/territories), sex (female), age (10–54 years), year (1990–2021), and Socio-Demographic Index (SDI) levels from GBD 2021. The SDI, ranging from 0.005 to 1, reflects the level of social development in a country or region, incorporating factors such as total fertility rate, per capita income, and average years of schooling (14). It is categorized into five levels: low, low-middle, middle, high-middle, and high.

The study focused on women aged 10–54 years, divided into nine age groups: 10–14, 15–19, 20–24, 25–29, 30–34, 35–39, 40–44, 45–49, and 50–54 years. In the GBD study, MHD include gestational hypertension (onset after 20 weeks gestation), pre-eclampsia, severe pre-eclampsia, and eclampsia. Chronic hypertension (onset prior to pregnancy or before 20 weeks gestation) was excluded unless superimposed pre-eclampsia or eclampsia developed. MHD are classified under codes O11-O16.9 in the International Classification of Diseases, 10th Edition (ICD-10).

### 2.2 Data analysis

#### 2.2.1 Statistical analysis

The estimates in this study were reported as rates per 100,000 individuals to compare regional trends in the incidence, MMR, and DALYs of MHD. Each rate was accompanied by a 95% UI, defined by the 2.5th and 97.5th percentiles of 1,000 ordered samples. These samples integrated statistical analysis from multiple sources, including input data, measurement error adjustments, and residual non-sampling error estimates, to account for potential uncertainties (13).


$$\mathrm{Age}\;\text{standardised rate}=\frac{\sum_{i=1}^{A}a_{i}w_{i}}{\sum_{i=1}^{A}w_{i}}$$

where $a_{i}$ is the age specific rate and $w_{i}$ is the weight in the same age subgroup of the chosen reference standard population (in which *i* denotes the $i^{\mathrm{th}}$ age class) and *A* is the upper age limit.

Disease indicators in the GBD database were calculated using the total population as the denominator. However, this approach can be skewed by peaks in female fertility, necessitating adjustments to the age-specific rates for greater accuracy and applicability. To convert raw disease indicators to rates per 100,000 live births, the age-specific fertility rate (ASFR) from the GBD 2021 database was utilized (15).


$${\text{Incidence rate}}_{\mathrm{age}-\text{specific}}=\frac{{\text{Incidence rate}}_{\mathrm{age}\;\text{group}}}{\text{ASFR}}$$

$${\text{DALYs rate}}_{\mathrm{age}-\text{specific}}=\frac{{\text{DALYs rate}}_{\mathrm{age}\;\text{group}}}{\text{ASFR}}$$

Graphs were generated to illustrate the global and regional distribution and trends in the burden of MHD. All calculations were performed using Excel 2019 (Microsoft Corporation), and visualizations were created using R software with packages such as *ggplot2* and *segmented*.

#### 2.2.2 Frontier analysis

Frontier analysis evaluates the relationship between the burden of a specific disease and the level of sociodemographic development (quantified by the SDI), aiming to identify the potential for reducing this burden in different regions (16). By fitting models, the analysis determined the theoretically achievable minimum burden value (frontier value) for a given SDI. The Bootstrapping loop method was then used to calculate the gap between the actual observed values and the frontier values across regions, assessing the associated uncertainty.

#### 2.2.3 Decomposition analysis

Decomposition analysis quantifies the contributions of aging, epidemiological changes, and population growth to disease trends (17). It calculates the impact of each factor on the overall trend and assesses their role in either increasing or decreasing disease burden.

#### 2.2.4 Age, period, and cohort model

An APC model was used to estimate the independent effects of age, period, and birth cohort on the incidence and MMR rates of MHD. Maternal age was divided into nine groups (from 10–14 to 50–54), and six consecutive 5-year periods (1992–1996 to 2017–2021) were selected. The period 2002–2006 and the 30–34 years were established as reference categories. Age-specific live birth data from the GBD 2021 database was used as the reference population. Data processing was performed using the APC Web Tool^2^ and R software (version 4.4.3).

#### 2.2.5 Slope index of inequality and concentration index

To accurately quantify and evaluate health inequalities, this study utilized two established measures: the Slope Index of Inequality (SII) and the Concentration Index (CI). These indicators, respectively, captured absolute and relative differences in health outcomes across socioeconomic groups (17).

The SII was derived from the SDI ranking and represented the absolute disparity in health outcomes between the most and least advantaged socioeconomic groups. Larger absolute values of the SII indicate greater inequality in health outcomes. The CI measured the relative distribution of health outcomes across the population by constructing a concentration curve. The CI ranges from −1 to 1, with positive values indicating that health outcomes are more concentrated among individuals with higher socioeconomic status, while negative values suggest a greater concentration among those with lower socioeconomic status.

#### 2.2.6 Average annual percentage changes

The calculation of average annual percentage changes (AAPCs) and their 95% CIs is a crucial method for analyzing temporal changes in time-series data. AAPCs are determined using a segmented linear model, which identifies breakpoints to reflect the average rate of change in a variable over specific periods. Regression analysis of the connecting points determines the direction and magnitude of the trend (18).


$$\text{AAPC}=\{\exp(\frac{\sum w_{i}b_{i}}{\sum w_{i}})-1\}\times 100$$

$b_{i}$ is the slope coefficient for the $i^{\mathrm{th}}$ segment with i indexing the segments in the desired range of years, and $w_{i}$ is the length of each segment in the range of years.

#### 2.2.7 Estimated annual percentage changes

Estimated annual percentage changes (EAPCs) are utilized to analyze trends in morbidity and maternal mortality by quantifying changes in age-standardized rates (ASRs) over time. EAPCs are computed using a generalized linear model, assuming Gaussian-distributed ASRs and linearity on a logarithmic scale, which represents a constant rate of change (19).


Y=∝+βX+ε

EAPC=(exp(β)−1)×100

*Y* refers to In(ASR) , *X* the calendar year, and ε the error term. Based on this formula, *β* represents the positive or negative ASR trends.

## 3 Results

### 3.1 Global trend

**Figure 1:**
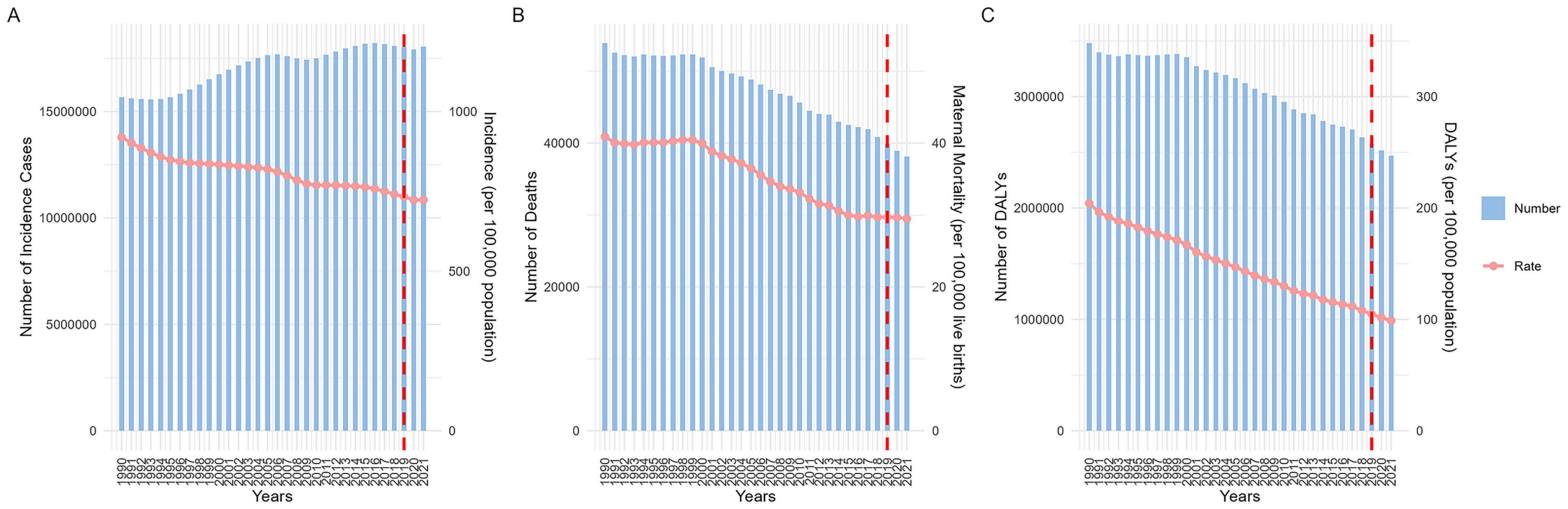
Global disease indicators: changes in quantity and rates from 1990 to 2021. (**A**: incidence; **B**: maternal mortality ratio; **C**: DALYs. DALYs, disability-adjusted life years).

Between 1990 and 2021, the number of maternal hypertensive disorders (MHD) cases increased annually, reaching a total of 18,050,085.26 (95%UI 15,356,123.72 to 18,050,085.26). Despite this rise, the incidence declined, decreasing from 919.34 (95%UI 759.23 to 1130.15) per 100,000 population in 1990 to 723.53 (95%UI 615.54 to 862.59) per 100,000 population in 2021. The number of deaths attributable to MHD and the associated maternal mortality ratio (MMR) have also shown a gradual decline. By 2021, death reached 38,147.32 (95%UI: 31,879.04 to 46,096.18) per 100,000 population, whereas MMR dropped to 29.49 (95%UI: 24.70 to 35.36) per 100,000 live births. Similarly, disability adjusted life years (DALYs) associated with MHD have decreased over time. Since 2019, the incidence has stabilized. However, global trends in deaths, MMR, and DALYs associated with these conditions have continued to decline (Figure 1).

### 3.2 Maternal hypertensive disorders across age groups

**Figure 2:**
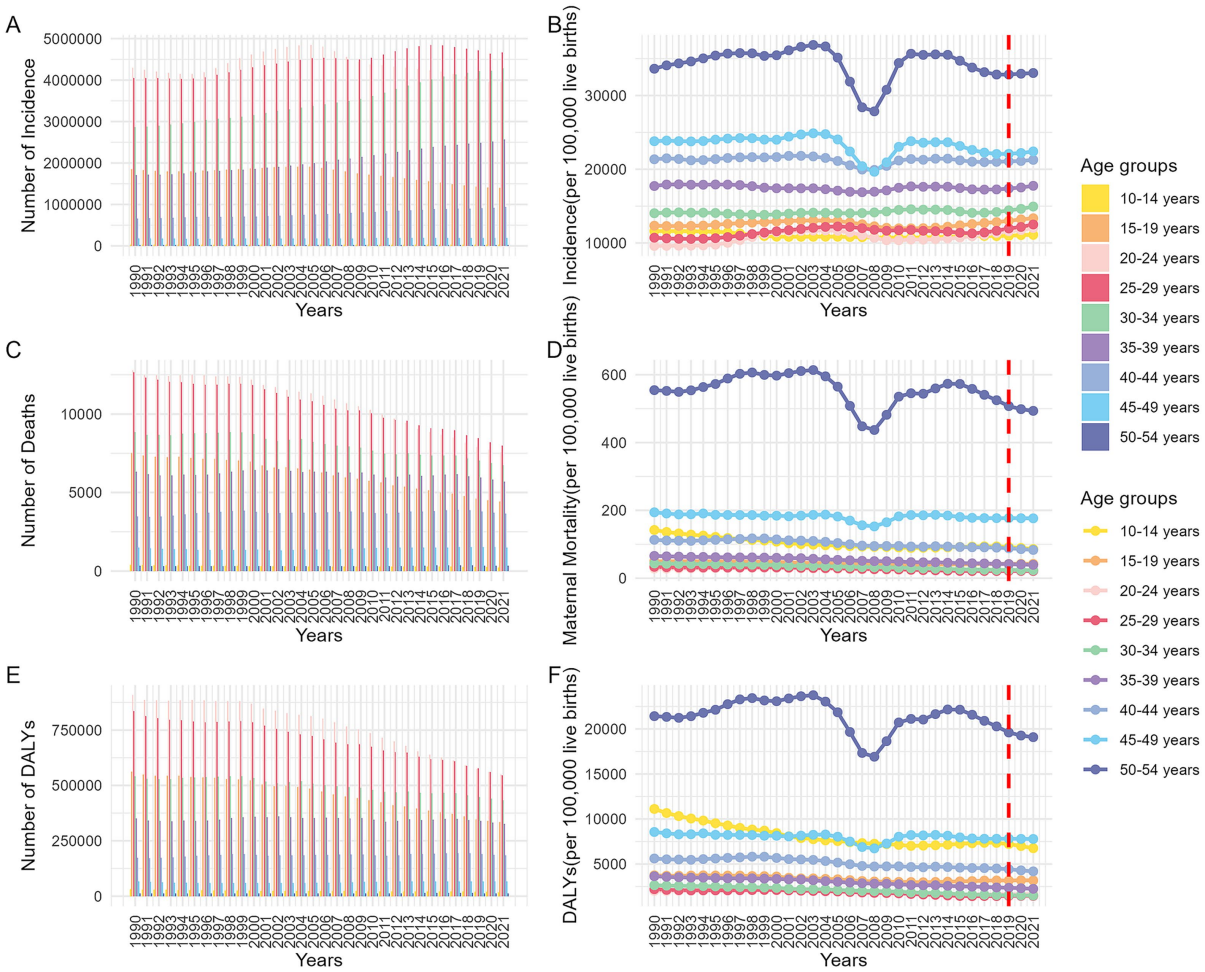
Trends in disease indexes (per 100,000 live births) across different age groups globally from 1990 to 2021. (**A**: number of incidence; **B**: incidence ratio; **C**: number of death; **D**: maternal mortality ratio; **E**: number of DALYs; **F**: DALYs ratio. DALYs, disability-adjusted life years).

Analysis of global MHD incidence from 1990 to 2021 revealed significant differences among age groups. Across all age groups, women aged 20–34 consistently reported the highest case numbers, while the 30–34 age group showed continuous growth in cases throughout the observation period. In terms of incidence rates, the 45–54 age group consistently maintained high levels. During the entire observation period, the incidence rate among pregnant women in this age group exhibited notable fluctuations between 2000 and 2010. In 2003, pregnant women aged 50–54 reached a peak incidence rate of 26,917.12 (95% UI: 23,057.84 to 31,099.14) per 100,000 live births, which dropped to 20,516.79 (95% UI: 17454.66 to 23627.64) in 2008, then rapidly rebounded to a plateau [25,998.63 (95% UI: 22,307.63 to 30,028.38)] in 2012, followed by a gradual decline. After 2019, incidence rates rose significantly across all age groups (Figures 2A,B).

Globally, MHD-related deaths decreased year by year, with the highest number of deaths occurring among pregnant women aged 20–29, while adolescent and older pregnant women had the lowest death counts. MMR across ages remained relatively stable, but MMR for pregnant women aged 50–54 was significantly higher than other groups, followed by those aged 10–14 and 40–44, while women aged 20–29 had the lowest MMR. Long-term trends showed notable fluctuations in MMR for the 50–54 age group around 2003 and 2008. From 2019 to 2021, MMR remained stable for most age groups, with only the 50–54 group showing a continued decline (Figures 2C,D).

This study assessed the impact of MHD on maternal health using DALYs. DALYs trends mirrored MMR patterns. Notably, the DALYs ratio for pregnant women aged 15–19 was nearly identical to that of the 40–49 group, both second only to the 50–54 group. The youngest group (10–14 years) saw a significant decline in DALYs after 1994, though rates remained higher than those of the 15–19 and 40–49 groups. The 50–54 group’s DALYs trends aligned with MMR changes but declined from 2019 to 2021 (Figures 2E,F).

### 3.3 Maternal hypertensive disorders in different SDI areas

This study demonstrated that, while global indicators for MHD were generally improving, the pace and extent of this improvement varied significantly across different regions. Developed areas consistently reported lower incidence compared to the global average, whereas less developed regions continued to experience higher MMR and DALYs due to MHD. Notably, by 2015, the incidence of MHD in Low-Middle SDI regions had fallen below the global average (Supplementary Figure 1).

**Figure 3:**
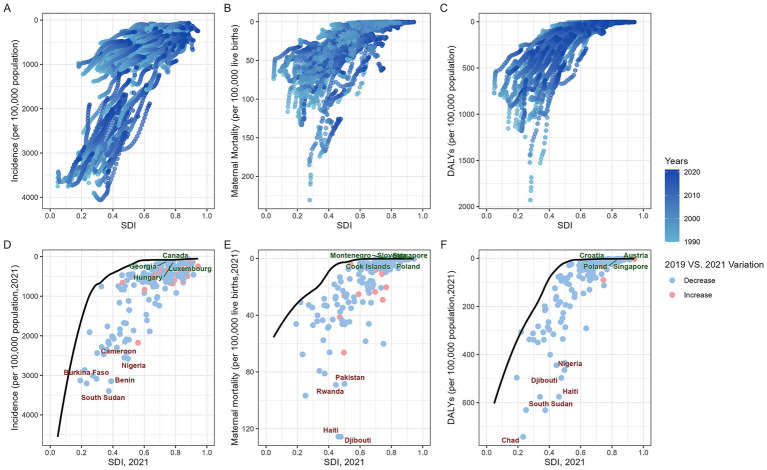
Global maternal hypertension disorders burden vs. SDI (1990–2021): Country trajectories and frontier shifts. (**A**: scatter plot of incidence; **B**: scatter plot of MMR; **C**: scatter plot of DALYs; **D**: frontier of incidence and changes between 2019 and 2021; **E**: frontier of MMR and changes between 2019 and 2021; **F**: frontier of DALYs and changes between 2019 and 2021. MMR, maternal mortality ratio; DALYs, disability-adjusted life years; SDI, socio-demographic index).

Globally, the disease burden of MHD tended to decrease as a country’s SDI increased (Figures 3A–C). Frontier analysis was conducted. The frontier, depicted as a solid black line, represents the optimal level of disease burden achievable at a given SDI level. The distance from this frontier, refer to as the effective difference, reflects the gap between observed outcomes and the best achievable outcomes under the same SDI level.

The incidence of MHD in countries at middle, high-middle, and high SDI levels (SDI > 0.6) was generally higher in 2021 compared to 2019. Countries such as Canada, Georgia, Hungary, and Luxembourg formed the frontier with the lowest incidence of MHD. In contrast, Cameroon, Nigeria, Burkina Faso, Benin, and South Sudan showed notable deviations from the expected levels for their respective SDI categories (Figure 3D). From the perspective of MMR, countries that stand out at different levels of the SDI included: the Cook Islands, Poland, Singapore, Slovenia, and Montenegro. Conversely, countries like Pakistan, Haiti, Djibouti, and Rwanda exhibited significant deviations from these optimal MMR levels (Figure 3E). Most countries experienced a decline in DALYs for MHD in 2021 compared to 2019. The countries with the lowest effective differences included: Austria, Singapore, Poland, and Croatia. In contrast, Nigeria, Haiti, Djibouti, Chad, and South Sudan continued to bear disease burdens that were markedly higher than what is expected for their respective SDI levels (Figure 3F).

### 3.4 Decomposition analysis and age-period-cohort model

**Figure 4:**
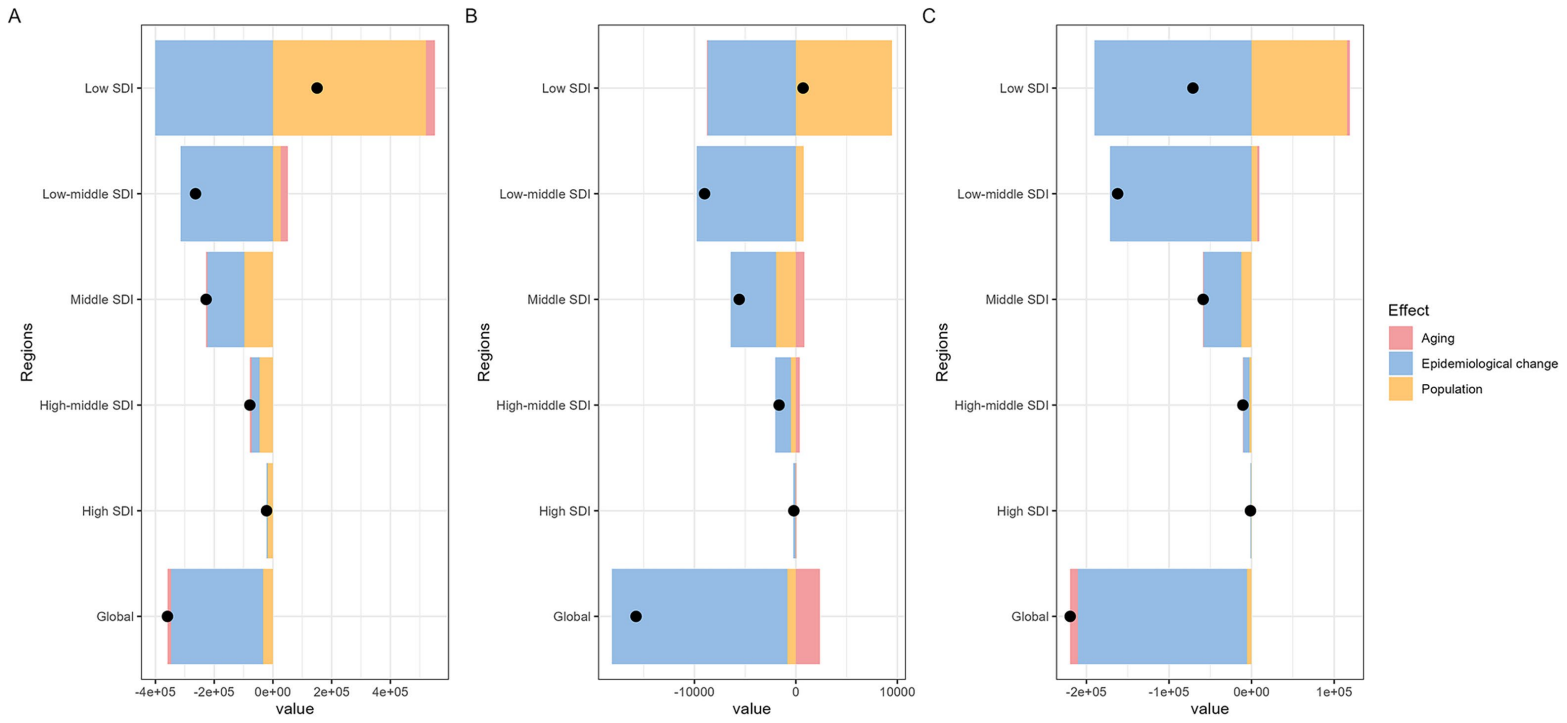
Global and five SDI regions decomposition analysis on incidence, maternal mortality ratio, and DALYs of maternal hypertension disorders (**A**: incidence; **B**: maternal mortality ratio; **C**: DALYs. DALYs, disability-adjusted life years; SDI, socio-demographic index).

Decomposition analysis shows that aging, epidemiological changes, and demographic factors contribute differently to MHD incidence, MMR, and DALYs (Figure 4). Demographic factors drive the increase in global MHD disease indicators, with the largest impact on incidence (Figure 4A). Aging reduces incidence and DALYs with a smaller effect, but contributes to the rise in MMR (Figure 4B). Epidemiological factors decrease MHD indicators, with the greatest impact on DALYs (Figure 4C). The effects of these factors vary across SDI regions. Demographic factors primarily drive the increase in disease indicators in all regions, while epidemiological factors play a major role in reducing disease indicators. In the Middle SDI region, aging contributes to the increase in MMR. These results highlight the different impacts of aging, epidemiological changes, and demographic factors on MHD.

**Figure 5:**
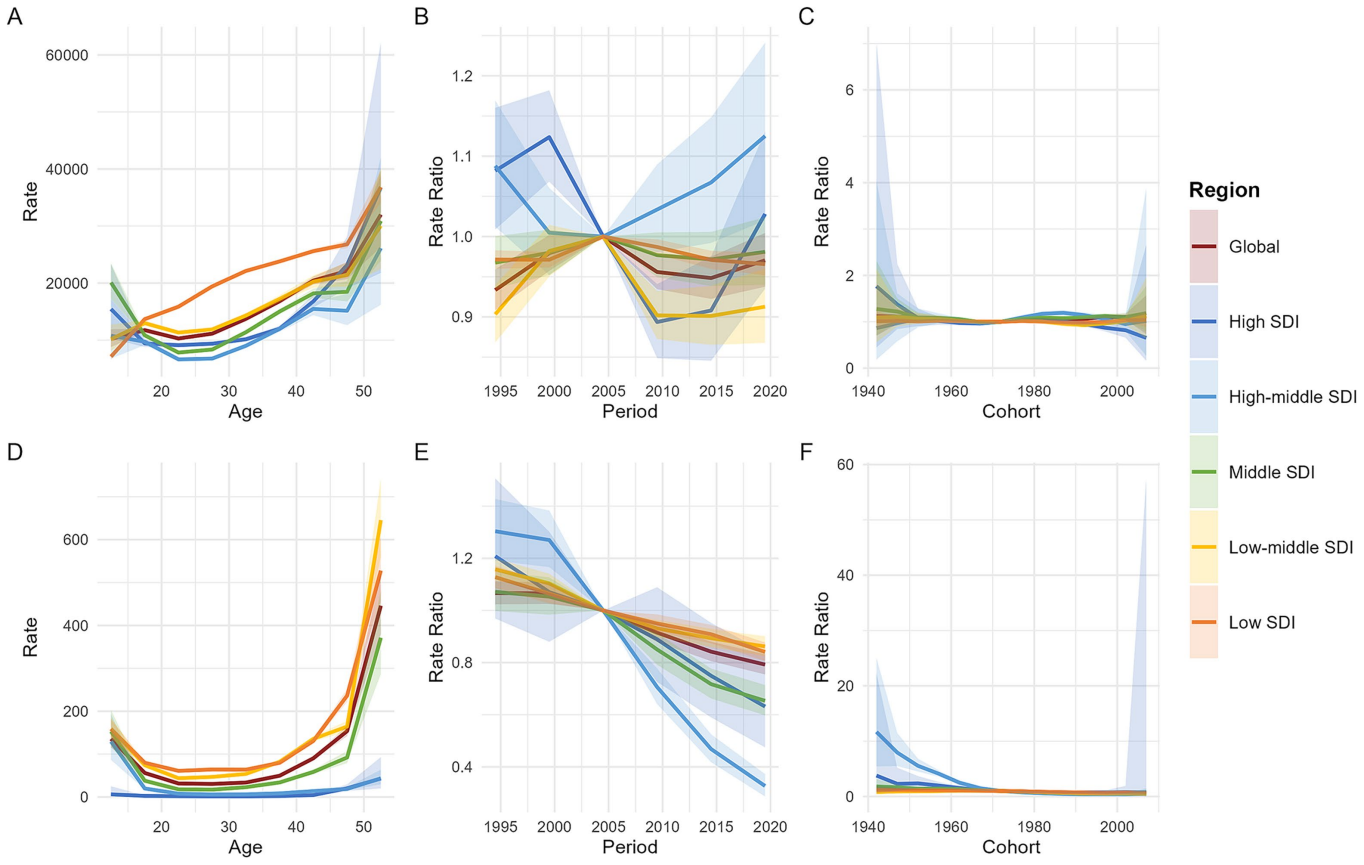
Age, period, and cohort effects on maternal hypertension disorder incidence and MMR by SDI quintiles. [**A**: the incidence (per 100,000 live births) after adjusting for age effects; **B**: the incidence after adjusting for period effects, with the reference period set to 2002–2006; **C**: the incidence after adjusting for cohort effects, with the reference cohort set to 1972; **D**: the maternal mortality ratio (per 100,000 live births) after adjusting for age effects; **E**: the maternal mortality ratio after adjusting for period effects, with the reference period set to 2002–2006; **F**: the maternal mortality ratio after adjusting for cohort effects, with the reference cohort set to 1972. Shaded areas represent the 95% confidence intervals. MMR, maternal mortality ratio; SDI, socio-demographic index].

Figure 5 displays the findings of the age-period-cohort (APC) model for both global and five SDI regions. The age effect is illustrated through longitudinal age curves, which depict the natural history of MHD incidence and MMR in relation to age. The period effect captures temporal variations, represented as relative risks across different time periods. Additionally, the cohort effect highlights change in relative risks across different birth cohorts.

From the perspective of age effects, both the global and Low-Middle SDI regions exhibit an N-shaped trend, characterized by an overall increase with age, but with a higher incidence rate in the 15–19 age group compared to the 10–14 and 20–24 age groups. In contrast, High-Middle SDI and Middle SDI regions show a U-shaped trend in incidence rates, marked by a decline until age 25, followed by an increase thereafter. The High SDI region also displays a U-shaped trend, with the turning point occurring at 17 years. The Low SDI region, however, demonstrates a continuous monotonic increase in incidence rates with age. After 17 years, the incidence rate in the Low SDI region is significantly higher than the global average and all other SDI regions; before 17 years, the Middle SDI region has the highest incidence rate. MMR associated with MHD exhibits a U-shaped distribution both globally and across all five SDI regions, with higher rates observed among mothers under 15 and over 50 years old. (Figures 5A,D).

Period effect analysis reveals that the global and different SDI regions exhibit markedly distinct temporal patterns in incidence and MMR. Using 2002–2006 as the reference period (RR = 1.0), the global incidence experienced fluctuating changes—initially rising, then declining, and rising again—overall maintaining a low risk level. The trends in incidence varied across SDI regions: High SDI regions showed an initial increase until 1999, followed by a decline from 2000 to 2009, and a subsequent rebound after 2010. High-middle SDI regions saw a decline before 2004, followed by a continuous rise, reaching the highest risk level among all SDI regions. Middle SDI and Low-middle SDI regions followed trends similar to the global pattern, which were characterized by three-phase fluctuations but maintained low risk levels. Low SDI regions experienced an increase from 1999 to 2004, followed by a sustained decline, also maintaining a low risk level. In stark contrast, MMR has consistently shown a downward trend globally across all SDI regions since the reference period, with High-middle SDI regions displaying relatively lower death risk levels. (Figures 5B,E).

After controlling for age and period factors, and using the 1972 birth cohort as the reference (RR = 1.0), the cohort effects on incidence remained relatively stable globally and across all SDI regions. In High SDI regions, the incidence among women born after 1992 showed a declining trend. Meanwhile, the risk of MMR due to MHD exhibited a continuous downward trend globally and in High, High-middle, Middle, and Low SDI regions. Specifically, the mortality risk remained relatively stable for birth cohorts after 1992 in High-middle SDI regions and after 1977 in High SDI regions. In contrast, in Low-middle SDI regions, the mortality risk increased with earlier birth years but began to decline gradually for cohorts born after 1962. (Figures 5C,F).

### 3.5 Cross-country social inequalities analysis

**Figure 6:**
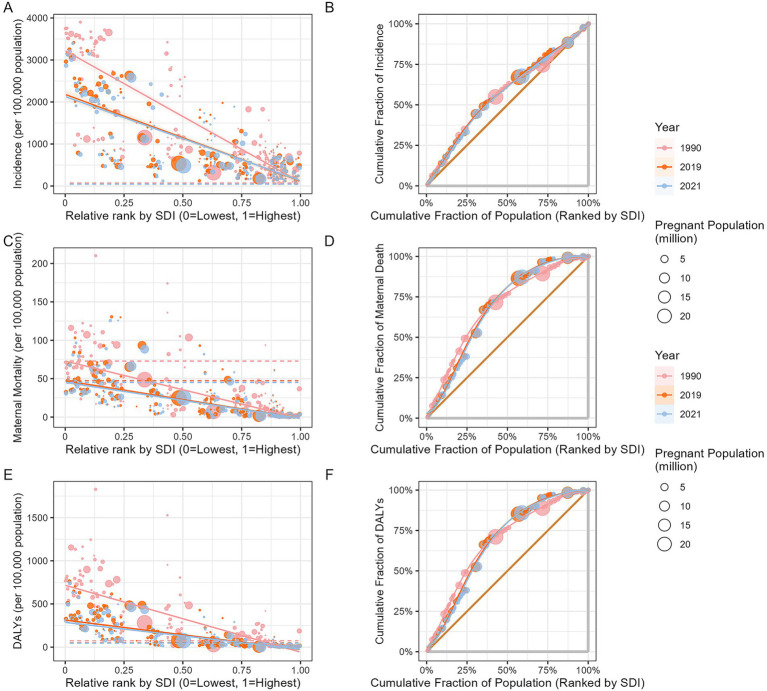
Slope index of inequality and concentration curves for the burden of maternal hypertensive disorders: 1990, 2019 and 2021. (**A**: health inequality regression curve for incidence; **B**: health inequality concentration curve of incidence; **C**: health inequality regression curve for MMR; **D**: health inequality concentration curve of MMR; **E**: health inequality regression curve of DALYs; **F**: health inequality concentration curve of DALYs. DALYs, disability-adjusted life years; MMR, maternal mortality ratio).

The SII and concentration curve were used to examine the absolute and relative inequalities in the burden of MHD in relation to the SDI. From 1990 to 2021, the SII for the incidence of MHD declined from −3131.05 (95% CI: −3372.28 to −2889.81) to −2003.35 (95% CI: −2184.75 to −1821.96). During the same period, the SII for MMR decreased from −74.74 (95% CI: −79.61 to −69.87) to −46.84 (95% CI: −50.88 to −42.80). For DALYs, the SII declined from −774.58 (95% CI: −817.27 to −731.88) to −312.45 (95% CI: −335.72 to −289.18). According to the concentration curve, the disease burden of MHD were more concentrated in poorer regions and populations. Although absolute inequalities had decreased, the concentration curves in 2021 shifted upward compared to 1990, indicating an increase in relative inequality (Figure 6).

Compared to 2019, the SII decreased in 2021. In 2019, the SII for incidence was −2063.24 (95% CI: −2254.04 to −1872.43), for MMR it was −49.08 (95% CI: −53.47 to −44.68), and for DALYs it was −341.47 (95% CI: −367.39 to −315.55). The concentration curves for 2019 and 2021 were similar, indicating that relative inequalities had remained unchanged (Figure 6).

### 3.6 Long-term and short-term dynamic changes in the disease burden of maternal hypertensive disorders

Table 1 presents the average annual percentage change (AAPC) worldwide and in five SDI regions over 1990–2021. Globally, the AAPC in the incidence of MHD decreased by −6.19% (95%CI: −6.61 to −5.78), while DALYs decreased by −3.27% (95%CI: −3.33 to −3.22), and MMR also declined by −0.40% (95%CI: −0.44 to −0.37). All disease indicators exhibited a downward trend, with incidence decreasing the fastest and MMR declining the slowest. Despite the overall global decline, significant differences were observed across SDI regions. Low SDI regions experienced much steeper declines compared to high SDI regions. In particular, the annual decline in incidence in low SDI regions was −29.46% (95%CI: −30.06 to −28.85%), approximately 24.2 times faster than the decline in high SDI regions, which was −1.22% (95%CI: −1.69 to −0.75%). Similarly, MMR in low SDI regions declined 22.8 times faster than in high SDI regions. For DALYs, the annual decline in low SDI regions was −13.64% (95%CI: −13.79 to −13.48%), a staggering 85.3 times faster than the decline in high SDI regions, which was only −0.16% (95%CI: −0.17 to −0.15%).

**Table 1:** AAPC of disease indexes from 1990 to 2021 by regional level.

Location	AAPC of incidence rate (95% CI)	AAPC of maternal mortality rate (95% CI)	AAPC of DALYs rate (95% CI)
Global	−6.19 (−6.61, −5.78)	−0.40 (−0.44, −0.37)	−3.27 (−3.33, −3.22)
High SDI	−1.22 (−1.69, −0.75)	−0.04 (−0.05, −0.04)	−0.16 (−0.17, −0.15)
High-middle SDI	−2.23 (−2.79, −1.67)	−0.28 (−0.30, −0.26)	−0.93 (−0.96, −0.90)
Middle SDI	−7.15 (−7.59, −6.72)	−0.36 (−0.40, −0.33)	−2.60 (−2.66, −2.53)
Low-middle SDI	−17.55 (−19.00, −16.10)	−0.83 (−0.89, −0.78)	−8.76 (−8.96, −8.57)
Low SDI	−29.46 (−30.06, −28.85)	−0.91 (−0.93, −0.89)	−13.64 (−13.79, −13.48)

AAPC, annual percentage change; DALYs, disability-adjusted life years; SDI, socio-demographic index.

The period from 2016 to 2021 was divided into two phases: pre-pandemic (2016–2018) and during the pandemic (2019–2021). The global incidence of MHD was steadily declining before the pandemic, with an EAPC of −1.13% (95%CI: −1.19% to −1.07%). However, the rate of decline slowed after the pandemic, potentially stabilizing at a plateau, with an EAPC of −0.67% (95%CI: −1.54 to 0.21%). In contrast, MMR declined at an accelerated rate of −0.37% (95%CI: −0.67% to −0.08%) during the pandemic compared to the pre-pandemic period. DALYs also showed an accelerated decline during the pandemic (Table 2).

**Table 2:** EAPC of disease indexes globally and across different regions in the periods of 2016–2018 and 2019–2021.

Regions	EAPC from 2016 to 2018			EAPC from 2019 to 2021		
	Incidence (95% CI)	Maternal mortality (95% CI)	DALYs (95% CI)	Incidence (95% CI)	Maternal mortality (95% CI)	DALYs (95% CI)
Global	−1.13 (−1.19,-1.07)	−0.11 (−0.8,0.59)	−2.53 (−3.45,-1.6)	−0.67 (−1.54,0.21)	−0.37 (−0.67,-0.08)	−2.78 (−3.02,-2.54)
High SDI	1.75 (1.74,1.77)	−3.27 (−3.98,-2.55)	−1.33 (−1.57,-1.09)	2.23 (0.33,4.18)	−5.07 (−5.63,-4.5)	−1.41 (−2.44,-0.37)
High-middle SDI	−1.69 (−1.91,-1.47)	−2.44 (−2.82,-2.06)	−5.23 (−5.59,-4.86)	−2.34 (−2.76,-1.92)	−1.98 (−3.03,-0.93)	−5.5 (−6.69,-4.29)
Middle SDI	−1.91 (−2.06,-1.76)	0.32 (0.15,0.49)	−3.28 (−3.66,-2.89)	−1.98 (−2.82,-1.13)	0.66 (0.42,0.9)	−3.58 (−4.5,-2.65)
Low-middle SDI	−2.39 (−2.48,-2.3)	−1.22 (−2.52,0.1)	−3.79 (−5.14,-2.42)	−0.94 (−3.14,1.3)	−1.51 (−1.63,-1.4)	−4.05 (−4.25,-3.85)
Low SDI	−1.97 (−1.98,-1.96)	−1.65 (−2.46,-0.83)	−3.61 (−4.38,-2.84)	−1.85 (−1.91,-1.79)	−2.16 (−2.36,-1.96)	−3.91 (−3.94,-3.87)

EAPC, estimated annual percentage change; DALYs, disability-adjusted life years; SDI, socio-demographic index.

Changes in the burden of MHD across different SDI regions revealed significant variations between the two periods. In high SDI regions, incidence increased annually before the pandemic and continued to rise during the pandemic, with an EAPC of 2.23% (95%CI: 0.33 to 4.18%). Conversely, in low-middle SDI regions, incidence declined significantly before the pandemic but slowed considerably during the pandemic, with a less pronounced trend [−1.22% (95%CI: −2.52 to 0.10%)]. In high-middle SDI regions, incidence decreased significantly before the pandemic, and this decline accelerated during the pandemic [−2.34% (95%CI: −2.76% to −1.92%)]. Regarding MMR, trends varied across SDI regions. In middle SDI regions, MMR increased slightly before the pandemic but accelerated during the pandemic [0.66% (95%CI: 0.42 to 0.90%)]. In high-middle SDI regions, MMR declined significantly before the pandemic, but the rate of decline slowed during the pandemic to −1.98% (95%CI: −3.03% to −0.93%). In high SDI regions, MMR continued to decline during the pandemic, with an accelerated rate of −5.07% (95%CI: −5.63% to −4.50%). Across all regions, DALYs showed an accelerated rate of decline during the pandemic (Table 2).

## 4 Discussion

Maternal hypertensive disorders (MHD) pose a substantial threat to maternal health, with inequalities in disease burden influenced markedly by temporal dynamics, geographic distribution, and demographic characteristics. These differences contribute to inequities in health outcomes at both national and global levels. This study systematically tracked the evolution of MHD from 1990 to 2021, offering a comprehensive analysis of trends across age groups and SDI levels. Furthermore, it assessed the short-term effects of the international public health emergency, namely the COVID-19 pandemic, on maternal health outcomes.

### 4.1 Trends in the burden of maternal hypertensive disorders under socio-demographic index differences: a dynamic analysis across SDI levels and age groups

Between 1990 and 2021, the global impact of MHD underwent complex changes. Although the total number of cases did not significantly decline, the incidence slightly decreased, and the overall health burden improved, measured by DALYs. This suggests that the disease’s impact on quality of life is gradually diminishing. However, progress in reducing the MMR stagnated since 2014. This divergence can be attributed to several factors. Early pregnancy screening, promoted by the United Nations Millennium Development Goals (MDGs) (20), standardized treatment guidelines from the International Society for the Study of Hypertension in Pregnancy (ISSHP) (21), and increased global investment in maternal healthcare (22) have collectively contributed to lowering the incidence and health burden of MHD. Conversely, challenges such as extreme weather events, pandemics, natural disasters, reduced healthcare budgets, and regional conflicts have hindered efforts to improve maternal health, leading to a plateau in MMR improvements (23).

Using the APC model to analyze the global disease burden of MHD, we found that adolescent and advanced-age pregnant women faced higher risks after controlling for period and cohort effects. For adolescent pregnant women, these risks mainly arise from the incomplete development of the reproductive system and hormonal fluctuations (24). Advanced-age pregnant women, on the other hand, face additional risks due to vascular aging, metabolic issues, and chronic diseases (25). The current trend of delayed childbearing further exacerbates these risks (26, 27).

From the perspective of period effects, the incidence of MHD among women worldwide showed periodic fluctuations. From 2000 to 2004, improved screening methods led to an increase in detected cases, driven by the United Nations’ MDGs which encouraged countries to enhance maternal care and identify high-risk cases earlier (20). The introduction of new diagnostic guidelines by the ISSHP in 2000 facilitated earlier detection (21), while heightened health awareness and increased prenatal check-ups also contributed to this rise (28). From 2005 to 2008, the incidence declined as healthcare funding increased (22, 29), improvements in community-based medical services (30), and treatment protocols became more standardized (21). However, advancements in assisted reproductive technology (ART) (31), and a rise in metabolic disorders (32), all of which are recognized risk factors for MHD caused a resurgence in incidence after 2014. Between 2003 and 2008, improvements in critical care practices (33) and increased availability of life-saving drugs, such as magnesium sulfate (34), contributed to a reduction in maternal deaths.

Through decomposition analysis, we further identified the key factors influencing the disease burden of MHD. The results indicated that demographic changes increase the incidence, and aging populations contributing to heightened risks of MMR. Epidemiological factors, however, were found to be the primary drivers in improving the overall disease burden. These factors include the implementation of public health policies that promote improvements in health systems and healthcare service quality, the expansion of health education, the adoption of behavioral intervention strategies, and enhanced management and control of high-risk pregnancies. Notably, significant disparities existed across regions of differing developmental levels. While population growth contributed to an increased disease burden across all regions, middle-SDI regions faced a distinct challenge due to the rising MMR associated with population aging. Differences in disease burden highlight variations in healthcare resources, policies, and socioeconomic conditions. A study examining the global burden of maternal and neonatal diseases from 1990 to 2019 found a strong association between increased disease burden and socioeconomic inequality (35). Similarly, research based on GBD 2021 data revealed a significant link between gestational hypertension and sociodemographic factors during the same period (36). Understanding these disparities is crucial for developing effective intervention strategies.

In High SDI regions, the decline in disease burden among with girls aged 10–14 and women over 35 was more significant. This improvement can be attributed to enhanced health awareness and robust social support systems, including comprehensive education and high-quality prenatal care (2). In High-Middle SDI regions, we observed a reduction in disease burden across all age groups, although the rate of improvement was slower compared to the High SDI regions. These findings underscored the importance of strengthening education and healthcare to address MHD. Additionally, developed regions benefit from advanced monitoring and diagnostic tools, which facilitate early detection and effective management of MHD, thereby reducing MMR and DALYs. For example, Denmark has made notable progress by expanding its obstetric centers and implementing national guidelines, ensuring consistent and high-standard care for severe pregnancy complications (37).

Research indicated that hypertensive disorders of pregnancy (HDP) and gestational diabetes mellitus (GDM) disproportionately affect women in low- and middle-income countries (25), aligning with the findings of this study. In middle SDI regions, women aged 40–54 years faced significant challenges: those aged 40–44 and 50–54 years exhibited the steepest incidence increases, while both MMR and DALYs showed steady rises in the 45–54 age group. Conversely, adolescents aged 10–14 showed notable improvements across all health metrics. This generational shift was likely attributable to successful WHO-backed sex education initiatives (38), such as Mexico’s eugenics movement in 1932 (39) and India’s Udaan project launched in 2006 (40). However, the growing health crisis among women of advanced maternal age highlights systemic shortcomings in middle-income healthcare systems. For instance, there are marked disparities in access to antenatal care between urban and rural areas in India (41). Additionally, in 2015, only 10% of women aged 35 and older in Indonesia had completed secondary education (42). These examples demonstrate how limited healthcare resources and educational disadvantages compound the challenges faced by older mothers. Notably, our decomposition analysis revealed that population aging emerged as a unique and significant factor contributing to the increase in MMR in Middle SDI regions. To address these issues, Middle SDI regions should prioritize education for older women, implement targeted maternal health programs, and enhance the detection of high-risk pregnancies. Increasing investment in healthcare infrastructure and improving early diagnosis of pregnancy complications would significantly enhance maternal and child health outcomes.

In Low-Middle SDI regions, older women exhibited similar disease patterns to those in Middle SDI areas, while adolescents bore a disproportionately heavy health burden. WHO data indicated that while global adolescent birth rate had decreased, it remained disproportionately high in poorer regions such as sub-Saharan Africa and Latin America, a trend linked to deep-rooted issues like child marriage, limited education, and inadequate access to contraception (43). For older mothers, risks are exacerbated by a combination of biological aging, frequent pregnancies, and limited healthcare resources (44). Low SDI regions reported a rising incidence of health issues across all age groups, with particularly sharp increases among 10–14-year-olds and women aged 50–54, highlighting the dual crisis affecting both young and older mothers. Surprisingly, MMR and DALYs declined most rapidly in these same groups. This paradox underscores the success of targeted health interventions, such as medical teams trained through realistic birth emergency simulations manage complications more effectively (45); Kenya’s community-based “Chamas for Change” program has improved survival rates for both mothers and infants (30); and upgrading healthcare facilities has encouraged more women to deliver safely in hospitals (46). Despite improvements in health programs in underdeveloped areas, their overall health statistics still lag significantly behind those of more developed regions.

### 4.2 The impact of the COVID-19 pandemic on maternal hypertension disorders and maternal health: challenges and responses across different regions

In the aftermath of COVID-19, the decline in incidence and DALYs of MHD notably slowed globally, while MMR remained relatively stable. Several factors may explain this trend. Disruptions in routine prenatal care diminished the effectiveness of disease management (47); COVID-19 infections, by inducing widespread inflammation, immune system imbalances, and blood clotting issues, exacerbated health challenges during pregnancy (48, 49); and regions with limited medical resources faced a compounded health crisis (50). Despite these challenges, many countries successfully mitigated these trends through targeted strategies. These included improving systems for monitoring high-risk pregnancies, refining antiviral treatments, and implementing remote programs for blood pressure management, all of which helped reduce serious complications (51).

High-SDI regions with advanced healthcare systems encountered significant challenges in managing MHD and reducing their health impacts (measured in DALYs) during the pandemic. Pregnant women with COVID-19 were particularly vulnerable to MHD, and changes in social dynamics and behaviors further heightened these risks (52). However, the stable MMR in these areas underscored the resilience of modern medical systems in crisis response. For example, Australia’s comprehensive implementation of the National Health Plan resources effectively sustained critical services such as primary care, specialist referrals, and maternity support (53).

During the pandemic, High-Middle SDI regions experienced a mixed impact on the prevention and control of MHD. While the incidence declined accelerated, the reduction in DALYs slowed, yet the overall trend remained positive. MMR, which had been increasing prior to the pandemic, stabilized. These regions mitigated the pandemic’s disruption to routine healthcare services by developing a flexible healthcare system, incorporating digital tools for prenatal advice and rapid-response systems for emergency maternity care, such as China’s online tiered medical care model (54). In contrast, Middle SDI regions faced significant systemic weaknesses. Their MMR, which had been declining, began to rise again, and improvements in DALYs slowed. This primarily stems from two key issues in MHD management: shortages in primary healthcare services and the overburdening of obstetric resources, akin to the widespread challenges in maternal healthcare access observed in Indonesia (55). These regional disparities highlight the critical importance of well-distributed healthcare resources in areas with High-Middle development levels to effectively manage public health emergencies.

In Low and Low-Middle SDI regions, the pandemic’s impact on health indicators manifested with a temporal delay. Our findings demonstrated a deceleration in MHD incidence, MMR, and DALYs. This observed slowdown predominantly stems from the inherent fragility of healthcare systems characteristic of these socioeconomic settings. For example, Haiti has long struggled with chronic shortages of clean water, sanitation, and medical supplies. Furthermore, the stigma surrounding COVID-19 during the pandemic further delayed patients from seeking essential care (56). Despite these challenges, core health metrics in these areas did not significantly deteriorate. This resilience can be attributed to international health assistance initiatives (57) and the effective management of limited local resources (56), both of which were crucial in maintaining essential healthcare services.

### 4.3 The differentiated characteristics of maternal health in the 21st century and global health policy responses

The 21st century’s most pressing maternal health challenge is the stark diversity and profound inequality that demand tailored strategies to ensure equitable care for mothers and children (58). Our analysis of health inequalities indicated a reduction in the absolute health inequality (SII) in the global burden of MHD. The findings suggested that regions with lower levels of socioeconomic development continued to bear a greater health burden. In contrast, the CI reflected relative inequality in relation to the SDI. The results further indicated that the relative distribution of the MHD burden remained more concentrated among socio-demographically disadvantaged populations. Wealthier nations, leveraging advanced education, top-tier medical services, and cutting-edge interventions, have significantly reduced the disease burden of MHD. However, poorer regions continue to struggle with limited resources and inadequate high-risk pregnancy care (59), exacerbated by insufficient global health financing (60), which further undermines already fragile systems. In these regions, older and teenage mothers face disproportionately higher risks, highlighting how developmental disparities translate into health inequalities.

The ongoing global public health emergency caused by the COVID-19 pandemic has contributed to the further widening of these disparities: low-resource countries grappled with overwhelmed hospitals and disrupted supply chains, leading to a surge in MHD cases as maternal care services faltered. In contrast, wealthier nations weathered the storm with resilient health systems and sustained funding (60). These disparities underscore the true strength of a nation’s health system—its policies, infrastructure, and crisis response. Alarmingly, over 3 billion people reside in countries where debt payments far exceed health spending (60), obstructing access to life-saving maternal care.

While the global MMR is declining, global policies must focus on underserved areas (44). Closing the gap requires dedicated funding, more efficient resource allocation, and targeted care for high-risk groups. In an era of frequent health emergencies, strengthening weaker health systems is not only a moral imperative but essential for global survival.

### 4.4 Strengths and limitations of this study

This study examined the trends and inequalities in the global disease burden of MHD between 1990 and 2021, using cross-stratified analysis by age and SDI. It also explored the short-term effects of the international public health emergency—namely, the COVID-19 pandemic. To account for the influence of fertility patterns on the disease burden, ASFRs were applied to adjust the MHD indicators for the target population of pregnant women. The adjusted results aligned closely with those obtained from the APC model, supporting the validity and consistency of our findings.

However, several limitations should be acknowledged. The GBD data were derived from disparate health information systems and reporting mechanisms across countries and regions, and variability in data collection methods may affected the accuracy and reliability of the results. In particular, data from low-income and less-developed regions, which primarily rely on sample surveys and national censuses, may suffered from missingness or bias, potentially influencing the overall estimation. Notably, data for age groups 10–14 and 50–54 years were extrapolated based on limited epidemiological evidence, and caution should be exercised when interpreting trends in these groups.

A notable limitation was the lack of detailed information on key risk factors for MHD in the GBD database—such as hypertension, advanced maternal age, multiple pregnancies, and gestational diabetes. The absence of these variables hampered a more comprehensive and mechanistic understanding of factors contributing to disease burden, and our interpretation largely remained based on decomposition analysis. Future supplementation of these critical indicators in the GBD database would greatly enhance the precision of MHD burden estimates and their clinical utility.

## 5 Conclusion

Despite significant progress in managing maternal hypertensive disorders, notable inequalities persist across different socioeconomic backgrounds and age groups. These differences were closely linked to socioeconomic factors and accessibility to medical resources. The emergence of public health emergencies exacerbated the management challenges in maternal health, particularly in resource-limited regions, highlighting the need for targeted strategies. Tailored prevention and management interventions that account for regional and demographic variations are crucial for safeguarding maternal health, promoting sustainable social development, and advancing global health equity and accessibility.

## Data Availability

Publicly available datasets were analyzed in this study. This data can be found here: https://ghdx.healthdata.org/.

## Footnotes

^1^ https://ghdx.healthdata.org/

^2^ https://analysistools.cancer.gov/apc/
