# Supplementary figures and images for: Tackling global inequalities in maternal hypertensive disorders: trends and the impact of public health emergencies, 1990–2021
Source: Front Public Health. 2025 Oct 13;13:1696754. doi: 10.3389/fpubh.2025.1696754 (PMC12554675; doi:10.3389/fpubh.2025.1696754)

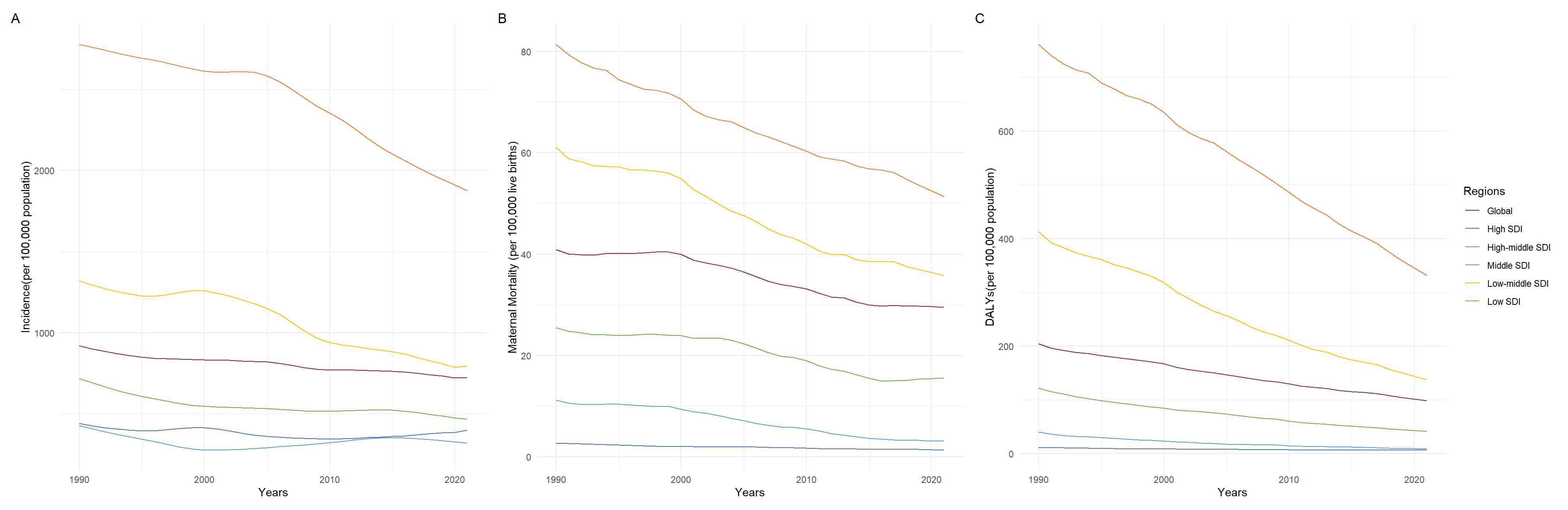

Supplement: Supplementary file 1 [file Image_1.TIFF]
